# The association between different timeframes of air pollution exposure and COVID-19 incidence, morbidity and mortality in German counties in 2020

**DOI:** 10.1186/s12940-024-01149-0

**Published:** 2024-12-26

**Authors:** Sophie Hermanns, Erika von Schneidemesser, Alexandre Caseiro, Susanne Koch

**Affiliations:** 1 Berlin School of Public Health, Charité – Universitätsmedizin Berlin, corporate member of Freie Universität Berlin and Humboldt Universität zu Berlin, Berlin, Germany; 2 Research Institute for Sustainability - Helmholtz Centre Potsdam, Potsdam, Germany; 3 Department of Anesthesiology and Intensive Care Medicine, Charité – Universitätsmedizin Berlin, corporate member of Freie Universität Berlin and Humboldt Universität zu Berlin, Campus Virchow-Klinikum, Augustenburger Platz 1, Berlin, 13353 Germany; 4 Department of Regional Health Research, University of Southern Denmark, Odense, Denmark; 5 Department for Anesthesiology, University Hospital Sjaelland, Nykøbing F., Denmark

**Keywords:** SARS-CoV-2, Air pollution, Intensive care medicine, Mechanical ventilation, Mortality, Nitrogen dioxide, Particulate matter, Short-term exposure, Long-term exposure, COVID-19

## Abstract

**Background:**

Ambient air pollution is a known risk factor for several chronic health conditions, including pulmonary dysfunction. In recent years, studies have shown a positive association between exposure to air pollutants and the incidence, morbidity, and mortality of a COVID-19 infection, however the time period for which air pollution exposure is most relevant for the COVID-19 outcome is still not defined. The aim of this study was to analyze the difference in association when varying the time period of air pollution exposure considered on COVID-19 infection within the same cohort during the first wave of the pandemic in 2020.

**Methods:**

We conducted a cross-sectional study analyzing the association between long- (10- and 2-years) and short-term (28 days, 7 days, and 2 days) exposure to NO_2_ and PM_2.5_ on SARS-CoV-2 incidence, morbidity, and mortality at the level of county during the first outbreak of the pandemic in spring 2020. Health data were extracted from the German national public health institute (Robert-Koch-Institute) and from the German Interdisciplinary Association for Intensive Care and Emergency Medicine. Air pollution data were taken from the APExpose dataset (version 2.0). We used negative binomial models, including adjustment for risk factors (age, sex, days since first COVID-19 case, population density, socio-economic and health parameters).

**Results:**

We found that PM_2.5_ and NO_2_ exposure 28 days before COVID-19 infection had the highest association with infection, morbidity as well as mortality, as compared to long-term or short-term (2 or 7 days) air pollutant exposure. A 1 μg/m^3^ increase in PM_2.5_ was associated with a 31.7% increase in incidence, a 20.6% need for ICU treatment, a 23.1% need for mechanical ventilation, and a 55.3% increase in mortality; an increase of 1 μg/m^3^ of NO_2_ was associated with an increase for all outcomes by 25.2 – 29.4%.

**Conclusions:**

Our findings show a positive association between PM_2.5_ and NO_2_ exposure and the clinical course of a SARS-CoV2 infection, with the strongest association to 28 days of exposure to air pollution. This finding provides an indication as to the primary underlying pathophysiology, and can therefore help to improve the resilience of societies by implementing adequate measures to reduce the air pollutant impact on health outcomes.

**Trial registration:**

Not applicable.

**Supplementary Information:**

The online version contains supplementary material available at 10.1186/s12940-024-01149-0.

## Background

**Fig. 1:**
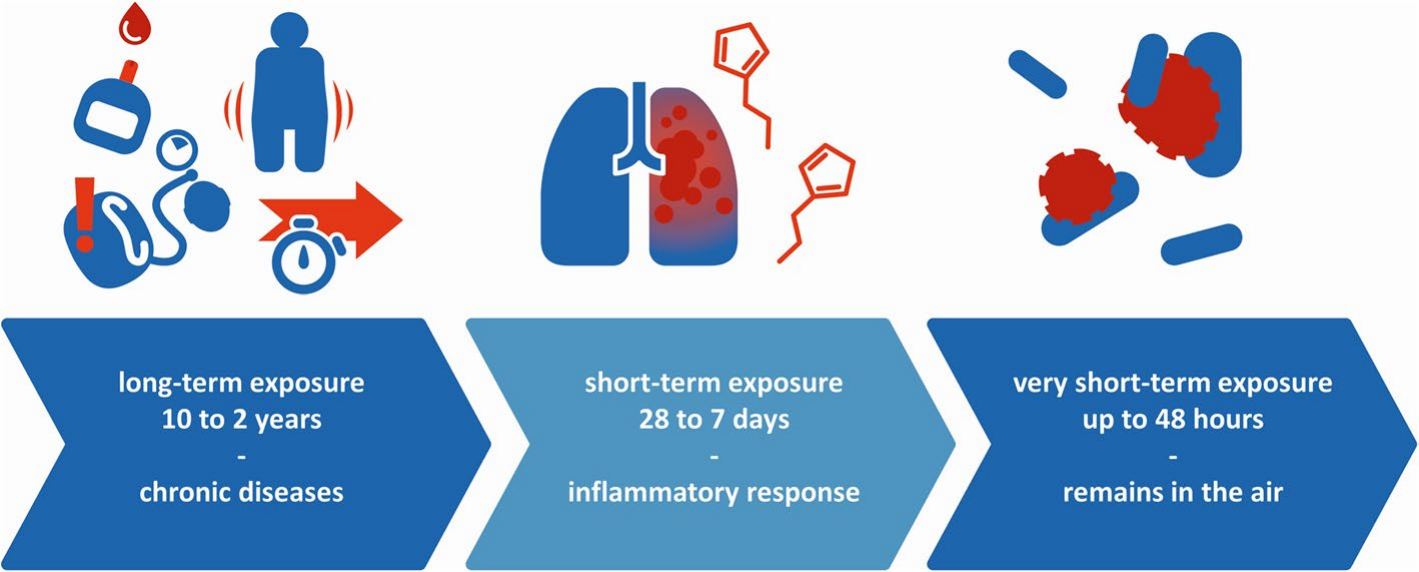
Air pollutant exposure can increase the vulnerability towards adverse COVID-19 health outcomes. By long-term (10 to 2 years) exposure the induction of chronic diseases are known risk factors for COVID-19 infection, morbidity and mortality. In the short-term (28 to 7 days) exposure, air pollutants might increase COVID-19 vulnerability by inducing an inflammatory response in the lung tissue and the human body. Finally, as SARS-CoV2 can adhere to small particulate matter in the air, this might also increase COVID-19 incidence within very short-term exposure up to 48 h

Ambient air pollution is a significant driver of morbidity and mortality in Germany. For the year 2018, the European Environment Agency attributed 63,000 premature deaths in Germany to particulate matter (PM) and 9,000 premature deaths to nitrogen dioxide (NO_2_) [1]. The Institute for Health Metrics and Evaluation estimated that air pollution was the 10th greatest health risk in Germany in 2019, with 29,252 attributable deaths or 3.05% of all deaths, while Lelieved et al. estimated that 11,000 deaths annually in Germany are attributable to road traffic emissions alone [2, 3]. Since the outbreak of the COVID-19 pandemic, a growing body of research has looked at the association between exposure to air pollutants and COVID-19 incidence, mortality and morbidity, showing that exposure to air pollution could affect COVID-19 morbidity and mortality through multiple potential mechanisms (Fig. 1) [4].

Over the long-term, exposure to air pollution increases the risk and severity of several chronic health conditions, including pulmonary, cardiovascular, and renal diseases, as well as obesity, diabetes, and cognitive decline, all of which are known risk factors for a more severe outcome in COVID-19 patients [5–8]. Upon inhalation air pollutants can cause oxidative stress and increase the concentration of reactive oxygen species (ROS), thereby damaging the epithelial lining fluid in the respiratory tract, which causes an inflammatory state in the lung tissue and thereby increases the risk for heightened inflammation during COVID-19 illness [9, 10]. Some research has indicated that over shorter time scales, viruses may be able to attach to PM and thereby stay airborne for a longer time period and distance, increasing the risk of SARS-CoV-2 transmission [11, 12].

An improved understanding of the timeframe of health impacts on COVID-19 incidence, morbidity and mortality from air pollutant exposure might help to elucidate the relative importance of the different pathophysiological mechanisms. Therefore, we aimed to analyze the timeframe of long-term (10 and 2 years) and short-term (28, 7 and 2 days) air pollutant exposure and the association with COVID-19 course in a single cohort, which is still scarce in the literature, since most studies on COVID-19 and air pollution have focused on either long-term exposure to air pollution, over several years preceding the pandemic, or on short-term exposure immediately before or during the period in which infections, hospital admissions and deaths occurred [4].

## Methods

The aim of this study was to analyze the effects of both long- and short-term exposure to NO_2_ and PM with a diameter of 2.5 μm or less (PM_2.5_) on COVID-19 disease burden at the county level (German: Kreise) for Germany during the first outbreak of the pandemic in spring 2020.

We conducted an observational county-based study built on the methods and data sources utilized in a study by Koch et al. (2022) [13]. Here we provide an analysis of the effects of long-term exposure, considering 10 years and 2 years period (2010–19 and 2018–19), a period where exposure to air pollutants might evoke chronic diseases. Additionally we estimate the effects of short-term exposure (28 days and 7 days), a typical period within which inflammation responses triggered by air pollutants might occur [10]. Finally, we included the time window were the SARS-CoV-2 virus might survive being attached to particulate matter, which is 48h [12]. We focused on COVID-19 incidence and mortality, but also leverage data from German hospitals to include admission to intensive care units and the need for mechanical ventilation of COVID-19 patients into the analysis.

Ethical approval was obtained from the ethical commission of the Charité (EA2/038/21; head: Prof. Dr. Kaschina). Patient consent was waived, because no individual patient data were collected and data analysis was performed anonymously.

### Setting and design

The unit of analysis is German counties, which corresponds to the Nomenclature of Territorial Unit for Statistics level 3 (NUTS-3). Most large cities and some smaller towns constitute their own counties. The first confirmed COVID-19 case in Germany was reported on January 27th. Germany’s Robert-Koch-Institute (RKI) counted 1,916,000 laboratory-confirmed COVID-19 cases and 33,000 deaths in 2020 [14]. To avoid bias in our dataset from COVID-19 spreading events, we limited our analysis to the first COVID-19 outbreak period (March 4th to May 16th) during which social distancing rules were implemented by the federal government and were therefore consistent over the whole country.

On March 15th, schools in Germany and national borders closed, followed by restaurants, shops and churches. Federal states started imposing social distancing rules from March 22nd onwards, limiting meetings between different households to two persons. Some states also restricted residents’ movement outside their homes. By April 15, these rules started to be lifted. Schools reopened on May 4th and borders started to be re-opened from May 15 [15]. Different regions were affected differently by the first wave, with high incidence in the large southern states of Bavaria and Baden-Württemberg and large cities, as well as cluster events during the February carnival festivities in the Rhine region. Many counties in the north and east were comparatively less affected during the first wave.

### Data sources

#### COVID-19 data

The German Interdisciplinary Association for Intensive Care and Emergency Medicine (DIVI) register tracks intensive care capacities and COVID-19 patient numbers in German hospitals [16]. Daily reporting to the register became mandatory for all hospitals on April 16, 2020. Data on COVID-19 patient-days on intensive care units and on mechanical ventilation were extracted for the period between April 16 and May 16, 2020. Using demographic data, we calculated the rate of patient-days per 100,000 residents. The Robert-Koch-Institute (RKI), Germany’s national public health institute, provides a public-access database of COVID-19 cases and deaths reported for each county by local public health offices [14]. All 401 counties in Germany reported cases and deaths from January onwards. However, only 396 counties reported to the DIVI-register and consistent data is only available from April 16th onwards [16]. The primary analysis of all outcomes is therefore limited to the DIVI reporting counties and the period from April 16th to May 16th, when most restrictions on social distancing, shops and schools began to be lifted. However, considering the entire first wave starting on March 4th, the day social restrictions were imposed in most of the country, only 18% of cases and deaths occurred in the shorter period starting on April 16th. Therefore, this longer period, which aligns with the RKI’s definition of the first wave, was used for secondary analysis.

#### Air pollution data

As in Koch et al. (2022), the APExpose dataset (version 2.0) was used to analyze the association between long-term exposure to air pollution and COVID-19 outcomes [17]. The data combines observed data from the European Environmental Agency’s Airbase database, with modelled global reanalysis data from the Copernicus Atmospheric Monitoring Service (CAMS) to create a complete dataset for all German counties for the period 2010—2019. The data includes parameters for nitrogen dioxide (NO_2_), nitrogen oxide (NO), ozone (O_3_), and particulate matter with an aerodynamic diameter smaller than 2.5μm and 10μm (PM_2.5_ and PM_10_), as well as three different scenarios (urban, rural, average). The parameters for NO_2_, NO, PM_10_ and PM_2.5_ are given as annual means while O_3_ is provided as the annual average of daily maximum 8-h. To analyze the effects of long-term exposure to air pollution, we calculated the means of each pollutant in each county over the ten-year period (January 2010 – December 2019) and the two-year-period (2018 – 2019) prior to the COVID-19 outbreak. NO and PM10 are highly correlated with NO2 and PM2.5, respectively, therefore no separate models were included in the main analysis.

To analyze the association of short-term air pollution exposure and COVID-19 outcomes, a new dataset was created, based on the same sources and methodology as APExpose at the daily time resolution. The data contains daily observations for the period from March 4th to May 16th, 2020, with values for NO_2_, O_3_ and PM_2.5_, averaged over the preceding 48-h, 7- day, and 4-week time periods of interest.

Temperature time series for the German counties, averaged at the same time resolutions as those used for the air pollution data, were obtained from the CAMS reanalysis.

### Demographic data and German index of social deprivation

The Federal Statistical Office of Germany provides data for each county on population size, area, and population distribution by age group and sex. Data from 2019 was used to calculate population density and the share of the population aged over 64 years, as well as the fraction of the population that is female. Population density is assumed to increase risk of transmission, and male sex and old age have been linked to increased risk of severe outcomes and death from COVID-19 [18, 19]. The German Index of Social Deprivation (GISD), developed by the RKI, is a measure of relative regional socio-economic disadvantage. The GISD indicators are selected to align with the concept of individual socio-economic status (SES) in social epidemiology, which combines education, occupation, and income dimensions. The index score is on a scale from 0 to 1. A higher score indicates more deprivation [20]. For each county, we calculated the mean GISD score between 2010 and 2019. Several ecological studies in Germany and other OECD countries have shown an association between income/social status and COVID-19 incidence. In the first wave of the pandemic, regions with higher income and education experienced higher incidence, possibly due to more international business and leisure travel [21, 22]. Studies found increased risk of mortality for socially deprived regions in Germany starting from the second wave of the pandemic, though findings for the first wave are less conclusive [23, 24]. Studies in the USA and UK have found increased risks for hospitalization and death for patients and regions with greater social deprivation [25–28].

### Statistics

The analysis has four outcome variables: new cases (incidence), new deaths (mortality), patient days on ICUs, and patient-days on mechanical ventilation. All outcomes were calculated as rates per 100,000 residents.

For the two long-term exposures, from 2010 to 2019 (ten years) and 2018 to 2019 (two years), air pollution and COVID-19 disease parameters were calculated as means per county. For short-term exposures, air pollution was calculated to provide averages over the preceding 2, 7 and 28 days for each date in a given county. The main analysis is limited to dates and counties for which data on patient-days on ICUs and mechanical ventilation were available through the DIVI-register, between April 16th and May 16th 2020.

Separate models for mean annual NO_2_ and mean annual PM_2.5_ were fitted for the ten- and two-year exposure periods and for the 48-h, 7-days and 4-weeks preceding each date. All models were adjusted to the following confounders: proportion of population aged over 65, the proportion of the population that was female, days between the first reported COVID-19 case and March 1st, population density, and the social depravation index score (Supplement Material Figure S1). Sensitivity analyses were conducted for tri-pollutant models with NO_2_, PM_2.5_ and O_3_ as combined exposures; Short-term models were also adjusted for temperature (daily mean dry temperature), as well as for weekdays only (excluding Saturdays, Sundays and Mondays from the model, since reporting of COVID-19 data from weekends could be delayed until Monday), and for the outcome parameter incidence and mortality the complete time period between March 4th and May 16th 2020 was also evaluated. Separate models were fit for each outcome, pollutant and exposure time-window.

As the annual mean O_3_ pollution (based on measurements of 8-h daily maximums) exceeded WHO-recommended thresholds in all counties between 2010 and 2019, but remained low during the first outbreak of the pandemic it was therefore excluded from the main analysis.

Negative binomial distributions were chosen due to overdispersion of the outcome variables. Because the control variables in the model operate at different scales (e.g. fractions of the population that are female were measured on a scale from 0 to 1; whereas e.g. population density had a larger range of values), the “scale” function in R was used to standardize them through centering and scaling, in order to improve comparability between variables. Since many counties experienced some days without new cases or deaths or patients on intensive care, the data used to model short-term exposure contained a high proportion of zeros (27 – 95%, varying by outcome). Therefore, zero-inflation was applied. This was not necessary for the long-term exposure models, as only a few counties reported zeros for outcomes aggregated over the entire study period (0 – 14%). To account for the repeat measurements at county-level and unmeasured factors affecting outcomes, random intercepts were fit for each county. Statistical analysis was conducted in R Statistical Software (version 4.3.1). Data processing was conducted with the tidyverse-package and models were fit with the MASS and glmmTMB packages [29–32].

## Results

### Disease outcomes

**Fig. 2:**
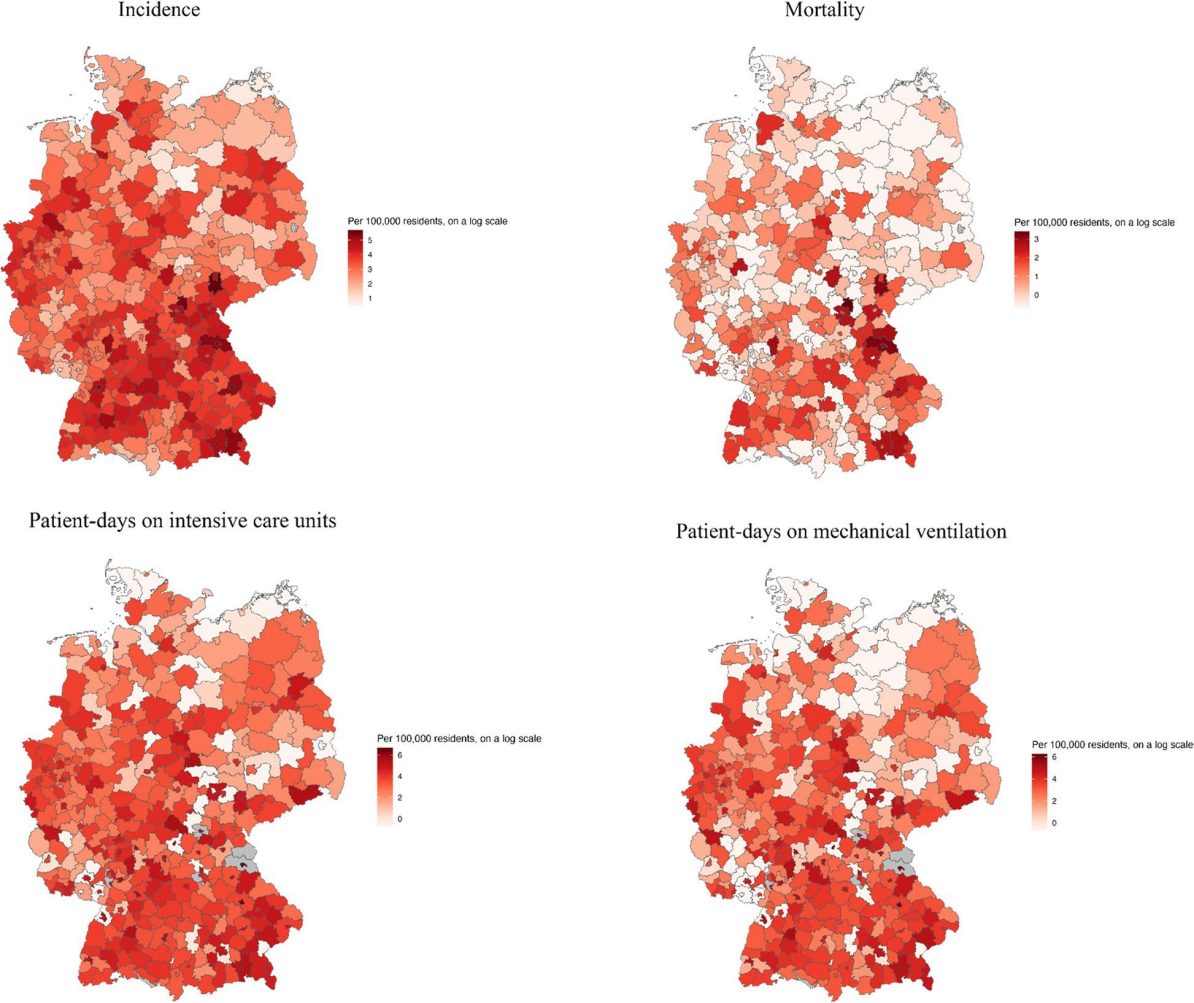
COVID-19 disease parameters per county for the period April 16th to May 16th 2020. Darker colors indicate higher values. Counties with missing data are marked in grey

Between April 16th and May 16th, in the 396 counties that reported to the DIVI-register, a total of 64,621 patient-days on ICUs and 46,234 patient-days on mechanical ventilation were recorded, as well as 31,660 new cases and 1,615 deaths. Between March 4th and May 16th 173,160 new cases and of 8,815 deaths were recorded. The average county reported 40.3 cases and 2.2 deaths per 100,000 residents between April 16th and May 16th, 54.4 patient-days on ICUs and 37.1 on mechanical ventilation per 100,000 residents (Figs. 2 and 4, Supplement Material Table S1).

#### Air pollution data

**Fig. 3:**
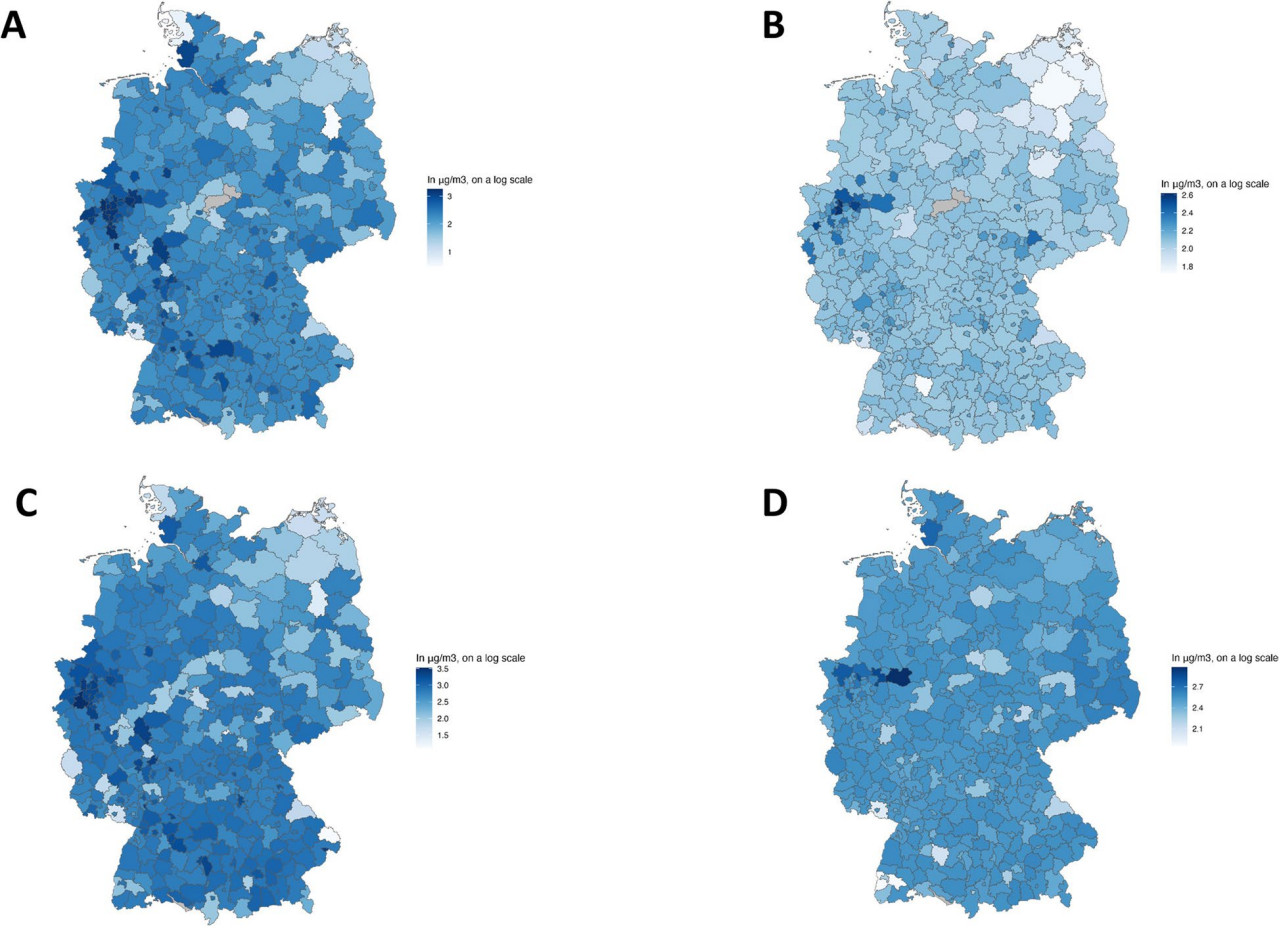
NO_2_-levels and PM_2.5_-levels per county as mean of the annual means for the period 16.04. – 16.05.2020 and over the last 10 years from 2010–2019. **A**) NO_2_-levels for the period 16.04. – 16.05.2020; **B**) PM_2.5_-levels for the period 16.04. – 16.05.2020; **C**) NO_2_-levels for the period 2010–2019; **D**) PM_2.5_-levels for the period 2010–2019. Darker colors indicate higher values

**Fig. 4:**
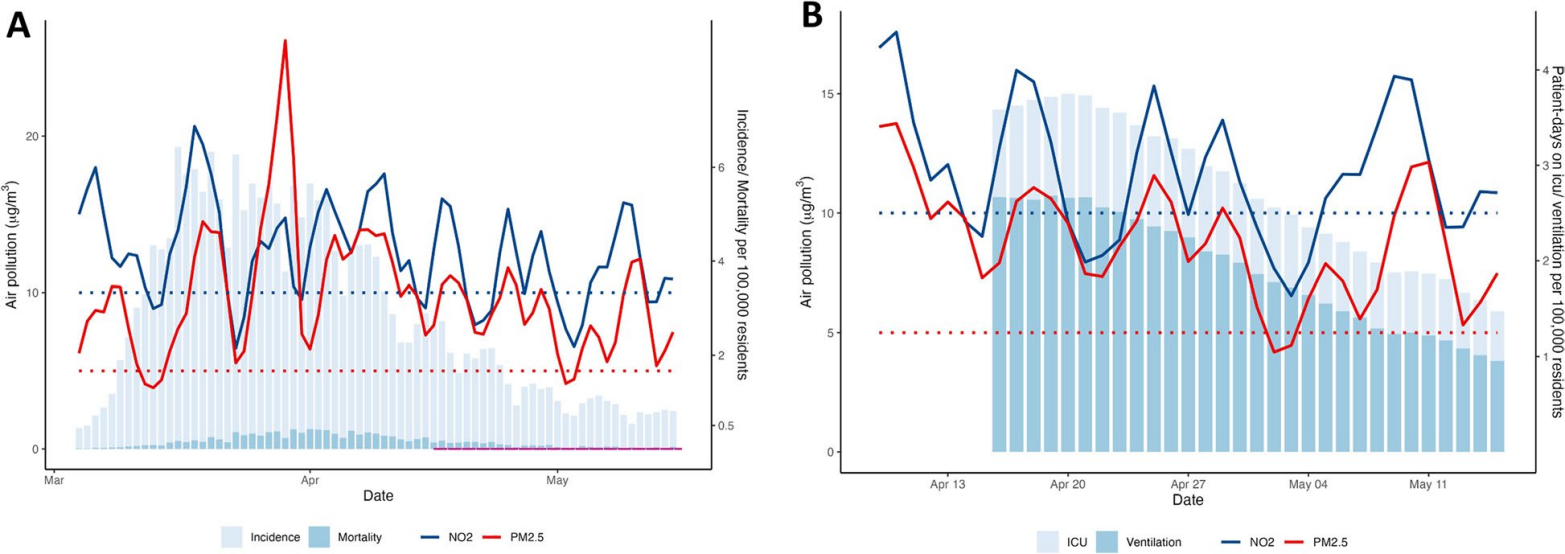
Outcome parameters and NO_2_-levels and PM_2.5_-levels over time. **A**) Incidence and mortality and NO_2_-levels and PM_2.5_-levels over time in March – May 2020. Pollution variables for a given date are given as average of the previous 48 h. The dashed colored lines are WHO-recommended thresholds annual average levels of NO_2_ and PM_2.5_. The pink line indicates the period for which data is available in the DIVI-register. B) Patient-days on ICU and on mechanical ventilation and NO_2_-levels and PM_2.5_-levels over time in April – May 2020. The dashed colored lines are WHO-recommended thresholds for annual average levels of NO_2_ and PM_2.5_

The data for analyzing short-term exposure contains 11,822 observations from 396 counties between April 16th and May 16th 2020. Data for the secondary analysis covering the entire first outbreak from March 4th to May 16th included 29,269 observations from 401 counties. Mean PM_2.5_ and NO_2_ pollution was slightly lower in 2018–19 (PM_2.5_: 12.5 μg/m^3^; NO_2_: 16.6 μg/m^3^) than in 2010–2019 (PM_2.5_: 13.1 μg/ m^3^; NO_2_: 17.8 μg/ m^3^) (Figs. 3 and 4, Supplement Material Table S1). The mean values of both pollutants averaged over two-, seven- and 28-day periods in the spring of 2020 were lower than the annual means from the preceding years (Fig. 3, Supplement Material Table S1).

### Long-term exposure models

**Fig. 5:**
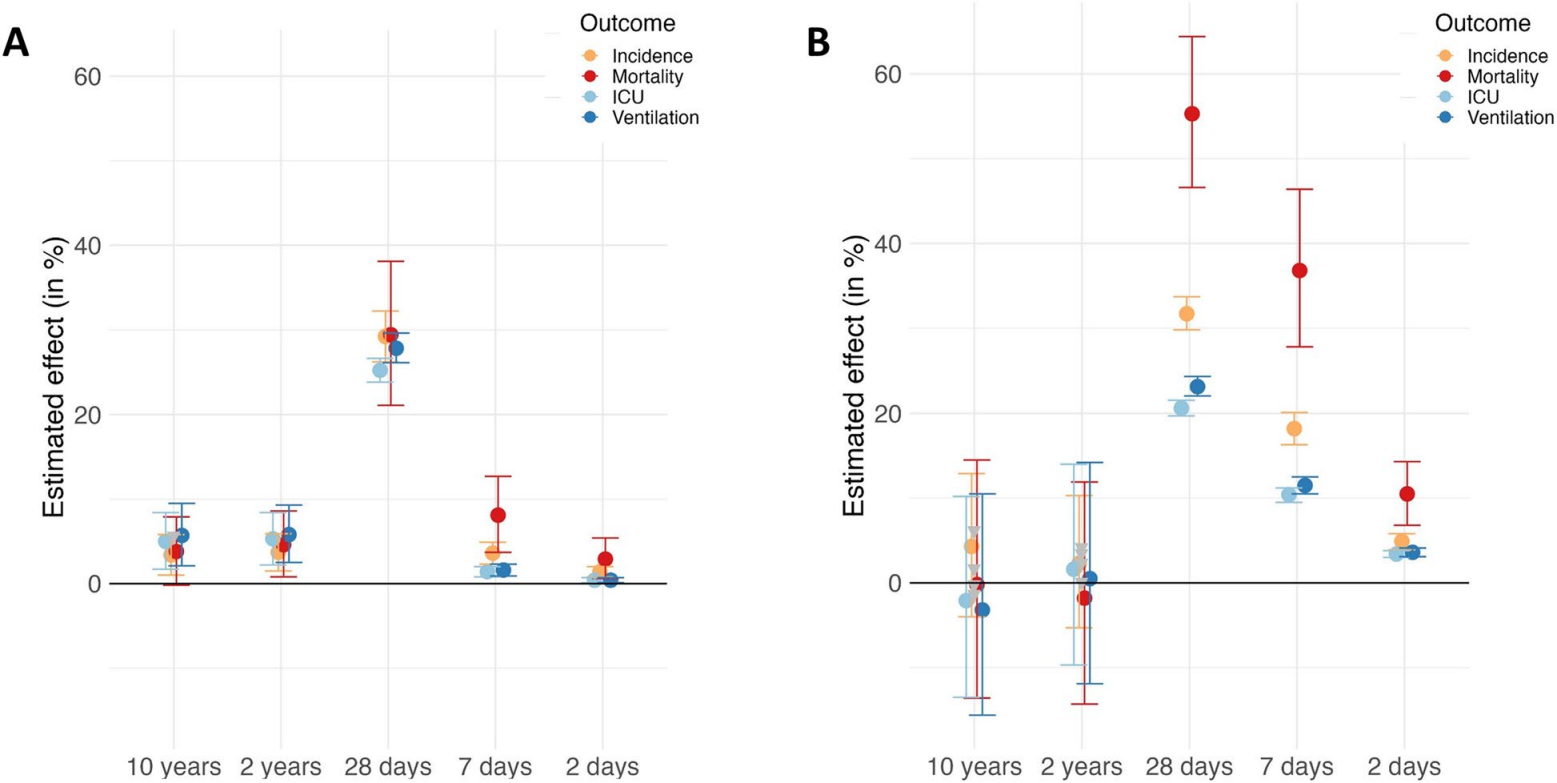
Effect estimates for the association of **A**) NO_2_ and **B**) PM_2.5_ on COVID-19 outcomes. Bars indicate 95% confidence intervals. Each estimate is derived from a separate, single-pollutant model adjusted to confounders (age over 65 years, sex, social depravation, population density and days between the first reported COVID-19 case and March 1.^st^)

In our analysis of the long-term single-pollutant exposure models for 10- and 2-year timeframes, PM_2.5_ has no statistically significant association with any outcome (Fig. 5, Table 1). In contrast, for NO_2_, the long-term single-pollutant exposure models show positive and statistically significant associations with all but one of the health outcomes examined (Fig. 5, Table 1). A 1 μg/m^3^ increase in mean annual NO_2_ between 2010 and 2019 was linked to a 3.4% (95% CI 1.010 – 1.058) increase in incidence, a 3.8% (95% CI 0.998 – 1.079) increase in mortality, a 5% (95% CI 1.017 – 1.084) increase in patient days on ICU, and a 5.7% (95% CI 1.021 – 1.095) increase in additional patient-days on mechanical ventilation. Similar results were seen for the 2-year timeframe, with the exception of mortality, where the increase of 3.8% was not statistically significant (95% CI 0.998–1.079, *p* = 0.053) (Table 1). The effect estimates for the exposure timeframe 2018–19 are of a similar magnitude and also yield a statistically significant estimate for mortality.

**Table 1:** Single-pollutant exposure models for PM_2.5_ and NO_2_ long-term (10 and 2-years) and short-term (28, 7 and 2 days) timeframes

		**NO**_**2**_ **April 16th to May 16th 2020**			**PM**_**2.5**_ **April 16th to May 16th 2020**		
**Outcome**	**Timeframe**	**Estimate (odds ratio)**	**95 CI**	*P*—**value**	**Estimate (odds ratio)**	**95 CI**	*P*—**value**
**Incidence**	10 years	1.034	1.010—1.058	0.0030	1.043	0.960—1.129	0.3068
	2 years	1.037	1.015—1.059	0.0006	1.023	0.947—1.103	0.5486
	28 days	1.292	1.262—1.322	0.0000	1.317	1.298—1.337	0.0000
	7 days	1.036	1.023—1.049	0.0000	1.182	1.163—1.201	0.0000
	2 days	1.014	1.008—1.020	0.0000	1.049	1.039—1.058	0.0000
**Mortality**	10 years	1.038	0.998—1.079	0.0528	0.998	0.864—1.145	0.9793
	2 years	1.046	1.008—1.086	0.0114	0.982	0.857—1.119	0.7813
	28 days	1.294	1.211—1.381	0.0000	1.553	1.466—1.644	0.0000
	7 days	1.081	1.037—1.127	0.0000	1.368	1.278—1.464	0.0000
	2 days	1.029	1.006—1.054	0.0150	1.105	1.068—1.143	0.0000
**ICU**	10 years	1.050	1.017—1.084	0.0015	0.979	0.865—1.102	0.6999
	2 years	1.053	1.022—1.084	0.0003	1.016	0.903—1.140	0.7588
	28 days	1.252	1.238—1.266	0.0000	1.206	1.197—1.215	0.0000
	7 days	1.014	1.008—1.020	0.0000	1.104	1.095—1.112	0.0000
	2 days	1.004	1.001—1.007	0.0030	1.034	1.030—1.038	0.0000
**Ventilation**	10 years	1.057	1.021—1.095	0.0010	0.968	0.844—1.105	0.5997
	2 years	1.058	1.025—1.093	0.0003	1.005	0.881—1.142	0.9356
	28 days	1.278	1.257—1.300	0.0000	1.231	1.220—1.243	0.0000
	7 days	1.016	1.009—1.023	0.0000	1.115	1.105—1.125	0.0000
	2 days	1.004	1.001—1.007	0.0110	1.036	1.031—1.041	0.0000

Negative binominal distribution models were calculated, adjusted to the confounders age, sex, social depravation, population density and days between the first reported COVID-19 case and March 1st

### Short-term exposure models

In the models for short-term exposure to NO_2_ and PM_2.5_, a mean increase of 1 μg/m^3^ over the preceding 2, 7, or 28 days is associated with an increase in all COVID-19 disease parameters and all estimates are statistically significant at the 5%-level (Table 1). The effect sizes are generally highest for pollution averaged over 28 days preceding disease outcomes and are larger in the PM_2.5_-models than in the NO_2_-models. A mean increase of 1 μg/m^3^ in PM_2.5_ in the 7 preceding days was associated with an 18.2% (95% CI 1.163 – 1.201) increase in incidence and a 36.8% (95% CI 1.278 – 1.464) increase in mortality (Table 1). A mean increase of 1 μg/m^3^ in NO_2_ in the 7 preceding days was associated with a 3.6% (95% CI 1.023 – 1.049) increase in incidence and an 8.1% (95% CI 1.037 – 1.127) increase in mortality (Table 1). Figure 5 visualizes the effect estimates of PM_2.5_ and NO_2_ on COVID-19 outcomes. Each estimate is derived from a separate model.

For patient-days on ICU and on mechanical ventilation, the effects are also largest after 28 days, with increases of 1 μg/m^3^ in either pollutant associated with increases of over 20% for both outcomes. However, these results should be regarded as only indicative, as the models for both outcomes exhibited poor fit and high dispersion values, ranging from 94 to 109, with a median of 9 × 10^6^. The models’ random effects indicated substantial variability at the county level (mean variance = 1.83, mean standard deviation = 1.33). This suggests that there are distinct county specific patterns.

### Sensitivity analyses

Sensitivity analyses were conducted that included data from March 2020, employed a tri-pollutant model for NO_2_, PM_2.5_ and O_3_ as combined exposures, adjusted for daily mean temperature and for weekdays only (excluding Saturdays, Sundays, and Mondays) in the short-term models. The results of the sensitivity analyses were consistent with the single pollutant model findings (Supplement Material, Table S2 – S4).

## Discussion

In our model, the 28-day short-term exposure to elevated PM_2.5_ and NO_2_ levels have the highest association to COVID-19 infection, morbidity as well as mortality, as compared to long-term (10 and 2 years) or very short-term (2 or 7 days) air pollutant exposure. In detail, an increase of 1 μg/m^3^ in PM_2.5_ in the 28 preceding days was associated with an 31.7% increase in incidence and an 55.3% increase in mortality, as well as an increase in the need for ICU treatment and mechanical ventilation of 20.6 and 23.1% each. For NO_2_ an increase of 1 μg/m^3^ at 28 days before COVID-19 infection, increased the risk for all assessed COVID-19 outcomes by 25.2 – 29.4%.

In a systematic review of 139 studies on long-term exposure, Bhaskar et al. found that 127 reported statistically significant positive associations between air pollution and adverse COVID-19 health outcomes [4]. Carballo et al. reviewed 355 pollutant-COVID-19 estimates from 116 long- and short-term exposure studies and found that approximately half reported significant positive associations for incidence (52.7% of studies) and mortality (48.1% of studies), with a slightly lower rate for non-fatal severe outcomes (41.2% of studies) [33]. Similarly, a meta-analysis of studies using data from individual patients reported 66% higher odds of COVID-19 infection and 127% higher odds for severe non-fatal outcomes per additional 10 μg/m^3^ of PM_2.5_, in addition to a positive but statistically not significant association with mortality [34].

Overall, our findings are consistent with other studies that have found positive and statistically significant associations between air pollution and COVID-19. However, a detailed time-frame association between the different mechanisms by which air pollutants cause adverse health effects in COVID-19 pandemic is still unresolved.

Even though epidemiological studies do not provide insights into the mechanisms by which air pollutants cause adverse health effects, disentangling the time-frame of air pollutant impacts on health in epidemiological studies can help to rank the importance of each possible mechanism. These results are critical for providing insights into causality and about how best to avoid adverse health effects from PM_2.5_ or NO_2_ in future. As already mentioned in the introduction, there are three hypotheses as to the mechanisms by which air pollutants could causally contribute to elevated transmission or adverse health effects, which can be ordered in time. Over the long-term, air pollution contributes to an increase in chronic diseases, which results in a higher vulnerability to COVID-19 [35–37]. In the short-term, air pollutants exacerbate the inflammatory response and thereby are linked to a higher susceptibility and adverse course after COVID-19 infection [9]. Finally, on the very short-term (2 days or less), there is the hypothesis that COVID-19 viruses persist in the atmosphere longer by attaching to particulate matter, which could increase the transmission and thereby incidence of COVID-19 [11]. It should be acknowledged that the role of particulate matter in COVID-19 transmission is disputed in the literature.

An attempt to decompose long-term (2 years) and short-term (up to 11 days) particulate matter effects on COVID-19 excess deaths were also undertaken by Becchetti et al. [38] They found a positive and significant effect on the long-term and the short-term exposure, with an increase in COVID-19 mortality of up to 20% for each increase of 1 µg/m^3^ in PM_2.5_. This is comparable to what we found in this study, where an increase in COVID-19 mortality of 18.2% (7 days) and 36.8% (28 days) was observed.

A study from Delhi, India analyzed short-term (one to three weeks) air pollution exposure and found the highest association with COVID-19 cases and death at two and three weeks [39]. These results are in line with our results, since we also found the highest association at 4 weeks of air pollutant exposure to COVID-19 incidence and mortality. However, Singh et al. found a higher association between NO_2_ air pollution and the COVID-19 incidence and mortality than with PM_2.5_ [39]. One main difference between both studies is, that air pollutant concentrations are much higher in India on average than in Germany and Singh et al. analyzed data from one, big city. In spring of 2020, the mean PM_2.5_ concentration in Germany was 9.8 µg/m^3^, as compared to 88 µg/m^3^ in Delhi, and the mean NO_2_ were 12.5 µg/m^3^ and 35 µg/m^3^ in Germany and Delhi, respectively. These differences likely impacted the results.

A recent study from China examined the impact of short-term (one week and one month), as well as mid-term (3 month), exposure to ambient air pollutants on disease recovery from COVID-19. They found, that exposure to high PM_2.5_ and NO_2_ level at one month before COVID-19 infection is associated with a prolonged recovery [40]. These findings are well in line with our results, showing the highest association with PM_2.5_ and NO_2_ exposure one month before infection, even though they focus COVID-19 recovery.

A study from Mexico looked at the impact of long- (years) and short-term (2 weeks) exposure to PM_2.5_ on mortality from COVID-19 in an individual level data base. They found an association between both long-term and short-term exposure to PM_2.5_ on COVID-19 mortality, which is in line with our short-term results, but not with our long-term results, where we did not find an association between PM_2.5_ exposure and COVID-19 mortality [41]. Interestingly, they also found a positive association between aging and health impact from ambient air pollutant exposure [41]. This indicates, that the mean age of a study cohort should be taken into account when comparing results from different studies.

A study from California assessing the impact of short-term (4-weeks) and long-term (6-years) exposure on COVID-19 mortality, found the highest positive association with long-term PM_2.5_ and NO_2_ exposure, as compared to short-term exposure [42]. The findings differ from our results, as we found lower or no long-term association with PM_2.5_ or NO_2_ exposure, but a significant short-term impact. As they also included study periods after COVID-19 vaccination has taken place, they could also show that vaccination reduced the risk association between air pollutant exposure and COVID-19 mortality risk.

Within our analysis we found the highest positive association between COVID-19 mortality, morbidity and incidence with elevated PM_2.5_ and NO_2_ on the timeframe of 28 days and then followed by 7 days, which is most likely related to an increased impact of an elevated inflammatory state within the pulmonary system caused by air pollutants and by then dramatically increasing the vulnerability towards COVID-19 of the exposed person. This result could even be an underestimation, since in Germany, like most European countries, we saw significant reductions in NO_2_ (30%) and PM_10_-levels (10%) in April 2020, compared to previous years due to lockdown measures [43]. However, even at these reduced levels, we found the highest association on COVID-19 disease burden by short-term exposure of PM_2.5_ and NO_2_.

As mentioned above, the age of the analyzed cohort should be included in the analysis, as well as the baseline level of air pollutant concentration in the atmosphere, since the relationship between the air pollutant concentration and the health impacts are not linear.

### Limitations

The limitation of the model used in our study lies in the fact that in most cases people who are exposed to elevated air pollutant concentrations in the short-term are also generally exposed to elevated air pollutant levels on the long-term, making it difficult to distinguish the associations between long- and short-term exposure and COVID-19.

Regional variations in prevention measures such as social distancing regulations are not accounted for in the model. Data from public health offices is likely to be incomplete and dating of case notifications may not accurately reflect disease onset. For example, the data on cases and deaths has reporting-lags on weekends, when public health offices were closed. Data for Saturdays, Sundays and Mondays (when the weekend notifications were processed) therefore likely have systematic errors. Data on ICU occupancy and mechanical ventilation was collected primarily for resource coordination in a health emergency, not scientific purposes, and may contain systematic and unsystematic errors.

Some counties, especially those that include major cities such as Berlin, Hamburg, or Munich, are very large and heterogeneous. They encompass sub-populations that experience very high and very low levels of social deprivation, which skew very young and very old, live near traffic and in green suburbs. In this study, we could not differentiate between these sub-populations or capture complexities in pollution exposure through factors such as mobility. For example, the county Hochtaunuskreis has some of the lowest levels of pollution in Germany, but many residents work in nearby Frankfurt and Mainz, which have some of the highest levels of pollution. Similarly, not all patient-days on ICUs may be due to residents of the county in which the reporting hospital is located: three of the ten counties with the highest reported incidence do not have ICUs and residents who required intensive care and ventilation were therefore likely reported by hospitals in nearby counties.

Case numbers are likely to be an underestimate and do not account for asymptomatic cases. The database only included cases after the patients tested positive and tests in the spring of 2020 were limited to patients who showed symptoms. Associations modeled in this paper may indicate the effect of air pollution on the development of symptoms rather than on an increased risk of infections.

## Conclusion

Our findings contribute to the emerging literature on the link between COVID-19 and air pollution by showing a positive association between PM_2.5_ and NO_2_ on the incidence and mortality between April and May 2020 in Germany, with the strongest association one to four weeks after exposure to high pollution levels. These findings contribute to a larger evidence base on the negative effects of air pollution on population health. Not only would reductions in air pollution ease the burden of many chronic diseases on the German health system, improved air quality might also make populations more resilient against the now endemic SARS-COV-2 and similar infectious diseases in the future by reducing the inflammatory response in the population.

Given the limitations of our and other studies, future research should focus more on individuals to contrast and distinguish the effects of long- versus short-term and unveil the impact on the different mechanisms by which air pollutants cause adverse health effects. Further research using patient-level data or disaggregating population-data by age-groups, sex co-morbidities and vaccination status, etc. would mitigate the limitations of epidemiological cohort studies to some extent.

As we and others could confirm, a link between short-term air pollution and the incidence, disease course and mortality in COVID-19 exists; additional tools could be deployed during future health emergencies, such as limiting industrial production and car traffic, construction projects in population centers, as well as existing tools, such as indoor air filters, facial masks or lockdowns, could have additional justification.

## Supplementary Information

Supplementary Material 1.

## Data Availability

The Air Pollution Data are available at: APExpose_DE dataset in the form of an ASCII file: APExpose_DE__2010–2019.csv. DOI: https://doi.org/10.1038/s41597-021-01068-6. See Caseiro et al., (2021) for all further information associated to the generation of the dataset. Population data were open source data from the Robert Koch Institute https://www.rki.de/EN/Content/Health_Monitoring/Public_Use_Files/public_use_file_node.html*;* https://www.intensivregister.de/#/inde*; and from the Federal Statistical Office of Germany at* https://www-genesis.destatis.de/genesis/online*.*
