# Supplementary material for: The association between different timeframes of air pollution exposure and COVID-19 incidence, morbidity and mortality in German counties in 2020
Source: Environ Health. 2024 Dec 26;23:112. doi: 10.1186/s12940-024-01149-0 (PMC11670380; doi:10.1186/s12940-024-01149-0)
Supplement: Supplementary file 1 — Supplementary Material 1. [file 12940_2024_1149_MOESM1_ESM.pdf]

Supplement Material

	April - May 2020				2010 - 2019		2018 - 2019		April - May 2020	
	COVID-19 Disease Parameters				Air Pollutants					
**State**	**Incidence**	**Mortality**	**ICU**	**Mechanical ventilation**	**$PM_{2.5}$ (µg/m^3)**	**NO_2 (µg/m^3)**	**$PM_{2.5}$ (µg/m^3)**	**NO_2 (µg/m^3)**	**$PM_{2.5}$ (µg/m^3)**	**NO_2 (µg/m^3)**
**Baden-Württemberg**	53.6 (39.6)	2.7 (2.3)	78.7 (68.9)	52.9 (49.6)	12.8 (1.3)	19.6 (3.8)	12.1 (1.4)	18.4 (3.7)	8.1 (2.4)	12.3 (5.3)
**Bayern**	60.7 (49.1)	3.8 (5.1)	90.7 (125.0)	63.1 (87.0)	12.9 (0.6)	18.6 (3.4)	12.5 (0.8)	17.5 (3.8)	8.4 (2.4)	11.2 (4.4)
**Berlin**	33.6 (-)	1.4 (-)	74.3 (-)	59.6 (-)	16.7 (-)	20.0 (-)	13.7 (-)	18.4 (-)	8.4 (2.6)	13.6 (3.8)
**Brandenburg**	27.2 (23.1)	0.9 (1.1)	27.2 (35.2)	12.6 (13.8)	14.0 (1.4)	14.0 (3.4)	12.7 (0.8)	12.6 (3.6)	7.4 (2.5)	8.0 (2.8)
**Bremen**	82.5 (47.0)	3.3 (1.5)	66.4 (53.0)	38.5 (25.2)	13.4 (0.3)	21.8 (0.4)	12.8 (0.1)	20.0 (0.7)	8.2 (3.2)	13.4 (5.0)
**Hamburg**	36.4 (-)	3.1 (-)	82.0 (-)	67.5 (-)	14.1 (-)	23.5 (-)	12.9 (-)	22.9 (-)	8.1 (2.8)	16.9 (4.7)
**Hessen**	39.3 (33.4)	2.0 (3.4)	56.5 (44.1)	40.8 (35.7)	13.2 (0.5)	18.2 (6.1)	12.4 (0.9)	16.9 (6.5)	8.3 (2.6)	11.8 (6.7)
**Mecklenburg-Vorpommern**	6.1 (2.9)	0.1 (0.3)	6.1 (8.1)	3.7 (5.3)	12.6 (0.4)	10.5 (4.4)	12.0 (0.8)	10.0 (4.4)	7.1 (3.3)	6.6 (4.0)
**Niedersachsen**	21.1 (18.6)	1.3 (2.0)	27.1 (31.9)	18.3 (25.2)	12.6 (0.8)	15.6 (3.0)	12.2 (0.9)	14.6 (2.8)	8.0 (2.3)	9.6 (3.5)
**Nordrhein-Westfalen**	42.0 (26.1)	1.9 (2.1)	47.1 (31.1)	34.2 (26.9)	14.0 (1.3)	21.9 (4.4)	13.3 (1.5)	20.8 (4.8)	9.4 (3.1)	15.9 (7.2)
**Rheinland-Pfalz**	23.6 (20.2)	1.2 (1.7)	36.8 (47.4)	23.7 (36.5)	12.9 (1.1)	17.1 (5.4)	12.3 (1.1)	15.8 (5.5)	8.6 (2.6)	11.1 (5.6)
**Saarland**	31.8 (11.8)	2.7 (3.0)	46.2 (39.0)	21.4 (17.7)	13.1 (0.5)	17.7 (2.5)	12.5 (0.7)	16.3 (2.6)	8.3 (2.3)	11.0 (3.3)
**Sachsen**	23.3 (19.3)	1.1 (1.6)	39.5 (64.1)	23.0 (30.7)	13.5 (1.0)	16.4 (3.8)	12.7 (1.3)	13.9 (3.9)	8.0 (2.3)	9.6 (3.7)
**Sachsen-Anhalt**	14.8 (13.4)	0.6 (1.1)	17.7 (21.9)	10.0 (15.3)	13.7 (1.2)	15.8 (3.3)	12.5 (0.9)	14.4 (3.6)	8.1 (2.6)	10.2 (3.8)

	April - May 2020				2010 - 2019		2018 - 2019		April - May 2020	
	COVID-19 Disease Parameters				Air Pollutants					
**State**	**Incidence**	**Mortality**	**ICU**	**Mechanical ventilation**	**$PM_{2.5}$ (µg/m³)**	**NO_2 (µg/m³)**	**$PM_{2.5}$ (µg/m³)**	**NO_2 (µg/m³)**	**$PM_{2.5}$ (µg/m³)**	**NO_2 (µg/m³)**
**Schleswig-Holstein**	19.7 (18.4)	0.7 (1.4)	13.5 (13.1)	10.0 (10.5)	12.6 (0.8)	14.7 (3.4)	12.6 (1.1)	13.8 (3.7)	8.1 (2.6)	10.8 (5.0)
**Thüringen**	48.3 (75.5)	3.8 (7.8)	53.1 (64.6)	37.5 (49.6)	12.7 (1.2)	15.7 (4.5)	12.1 (1.3)	14.3 (4.3)	8.6 (2.6)	9.9 (3.7)
**Total**	**40.3 (40.4)**	**2.2 (3.7)**	**54.4 (76.2)**	**37.1 (54.0)**	**13.1 (1.1)**	**17.8 (4.7)**	**12.5 (1.1)**	**16.6 (4.9)**	**9.8 (4.3)**	**12.6 (6.0)**

Table S1: Disease outcomes and air pollution levels by state. All disease parameters are given as means per 100,000 residents for the entire period April 16th – May 16th 2020. $PM_{2.5}$ and NO_2 levels are proved as means of the annual means for the periods 2010 – 2019 and 2018 – 2019. For the period April 16th – May 16th 2020, the means of the 48-hour means per county were calculated. Standard deviations for all parameters are given in parentheses.

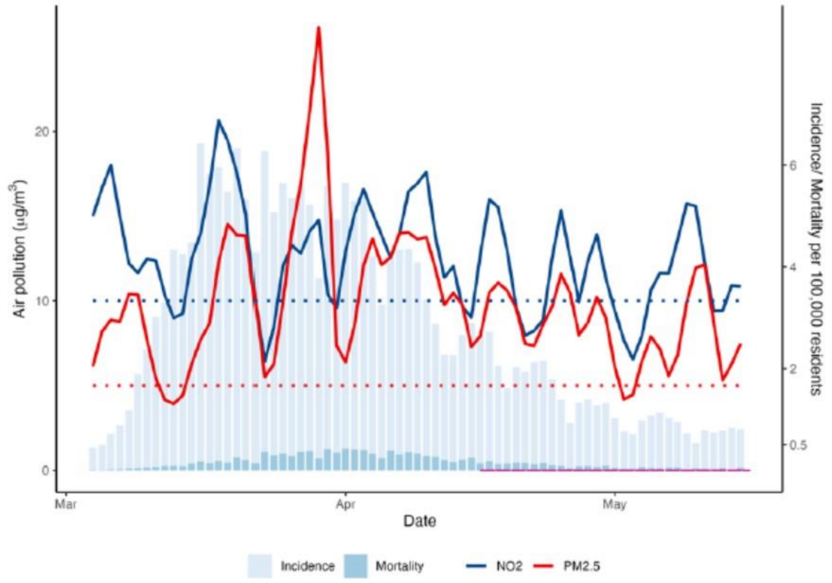

Figure 3: Incidence and mortality and NO_2-levels and $PM_{2.5}$-levels over time in March – May 2020. Pollution variables for a given date are given as average of the previous 48 hours. The dashed colored lines are WHO-recommended thresholds annual average levels of NO_2 and $PM_{2.5}$. The pink line indicates the period for which data is available in the DIVI-register.

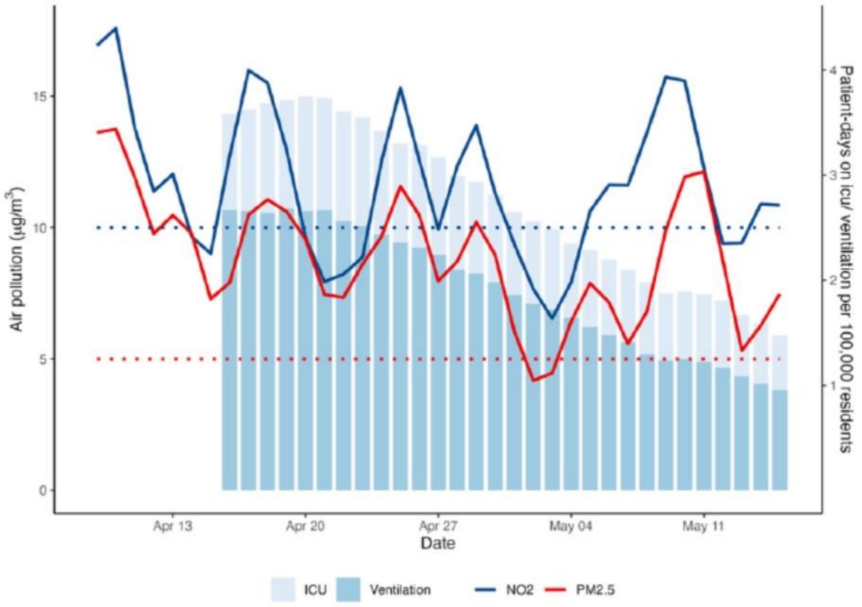

Figure 4: Patient-days on ICU and on mechanical ventilation and NO_2-levels and $PM_{2.5}$-levels over time in April – May 2020. The dashed colored lines are WHO-recommended thresholds for annual average levels of NO_2 and $PM_{2.5}$.

State	COVID-19 Disease Parameters				Air Pollutants					
	April - May 2020				2010 - 2019		2018 - 2019		April - May 2020	
	**Incidence**	**Mortality**	**ICU**	**Mechanical ventilation**	**$PM_{2.5}$ (µg/m^3)**	**NO_2 (µg/m^3)**	**$PM_{2.5}$ (µg/m^3)**	**NO_2 (µg/m^3)**	**$PM_{2.5}$ (µg/m^3)**	**NO_2 (µg/m^3)**
Baden-Württemberg	53.6 (39.6)	2.7 (2.3)	78.7 (68.9)	52.9 (49.6)	12.8 (1.3)	19.6 (3.8)	12.1 (1.4)	18.4 (3.7)	8.1 (2.4)	12.3 (5.3)
Bayern	60.7 (49.1)	3.8 (5.1)	90.7 (125.0)	63.1 (87.0)	12.9 (0.6)	18.6 (3.4)	12.5 (0.8)	17.5 (3.8)	8.4 (2.4)	11.2 (4.4)
Berlin	33.6 (-)	1.4 (-)	74.3 (-)	59.6 (-)	16.7 (-)	20.0 (-)	13.7 (-)	18.4 (-)	8.4 (2.6)	13.6 (3.8)
Brandenburg	27.2 (23.1)	0.9 (1.1)	27.2 (35.2)	12.6 (13.8)	14.0 (1.4)	14.0 (3.4)	12.7 (0.8)	12.6 (3.6)	7.4 (2.5)	8.0 (2.8)
Bremen	82.5 (47.0)	3.3 (1.5)	66.4 (53.0)	38.5 (25.2)	13.4 (0.3)	21.8 (0.4)	12.8 (0.1)	20.0 (0.7)	8.2 (3.2)	13.4 (5.0)
Hamburg	36.4 (-)	3.1 (-)	82.0 (-)	67.5 (-)	14.1 (-)	23.5 (-)	12.9 (-)	22.9 (-)	8.1 (2.8)	16.9 (4.7)
Hessen	39.3 (33.4)	2.0 (3.4)	56.5 (44.1)	40.8 (35.7)	13.2 (0.5)	18.2 (6.1)	12.4 (0.9)	16.9 (6.5)	8.3 (2.6)	11.8 (6.7)
Mecklenburg-Vorpommern	6.1 (2.9)	0.1 (0.3)	6.1 (8.1)	3.7 (5.3)	12.6 (0.4)	10.5 (4.4)	12.0 (0.8)	10.0 (4.4)	7.1 (3.3)	6.6 (4.0)
Niedersachsen	21.1 (18.6)	1.3 (2.0)	27.1 (31.9)	18.3 (25.2)	12.6 (0.8)	15.6 (3.0)	12.2 (0.9)	14.6 (2.8)	8.0 (2.3)	9.6 (3.5)
Nordrhein-Westfalen	42.0 (26.1)	1.9 (2.1)	47.1 (31.1)	34.2 (26.9)	14.0 (1.3)	21.9 (4.4)	13.3 (1.5)	20.8 (4.8)	9.4 (3.1)	15.9 (7.2)
Rheinland-Pfalz	23.6 (20.2)	1.2 (1.7)	36.8 (47.4)	23.7 (36.5)	12.9 (1.1)	17.1 (5.4)	12.3 (1.1)	15.8 (5.5)	8.6 (2.6)	11.1 (5.6)
Saarland	31.8 (11.8)	2.7 (3.0)	46.2 (39.0)	21.4 (17.7)	13.1 (0.5)	17.7 (2.5)	12.5 (0.7)	16.3 (2.6)	8.3 (2.3)	11.0 (3.3)
Sachsen	23.3 (19.3)	1.1 (1.6)	39.5 (64.1)	23.0 (30.7)	13.5 (1.0)	16.4 (3.8)	12.7 (1.3)	13.9 (3.9)	8.0 (2.3)	9.6 (3.7)
Sachsen-Anhalt	14.8 (13.4)	0.6 (1.1)	17.7 (21.9)	10.0 (15.3)	13.7 (1.2)	15.8 (3.3)	12.5 (0.9)	14.4 (3.6)	8.1 (2.6)	10.2 (3.8)
Schleswig-Holstein	19.7 (18.4)	0.7 (1.4)	13.5 (13.1)	10.0 (10.5)	12.6 (0.8)	14.7 (3.4)	12.6 (1.1)	13.8 (3.7)	8.1 (2.6)	10.8 (5.0)
Thüringen	48.3 (75.5)	3.8 (7.8)	53.1 (64.6)	37.5 (49.6)	12.7 (1.2)	15.7 (4.5)	12.1 (1.3)	14.3 (4.3)	8.6 (2.6)	9.9 (3.7)
**Total**	**40.3 (40.4)**	**2.2 (3.7)**	**54.4 (76.2)**	**37.1 (54.0)**	**13.1 (1.1)**	**17.8 (4.7)**	**12.5 (1.1)**	**16.6 (4.9)**	**9.8 (4.3)**	**12.6 (6.0)**

Table S1: Disease outcomes and air pollution levels by state. All disease parameters are given as means per 100,000 residents for the entire period April 16th – May 16th 2020. $PM_{2.5}$ and NO_2 levels are proved as means of the annual means for the periods 2010 – 2019 and 2018 – 2019. For the period April 16th – May 16th 2020, the means of the 48-hour means per county were calculated. Standard deviations for all parameters are given in parentheses.
